# Differential expression of midgut proteins in *Trypanosoma brucei gambiense*-stimulated vs. non-stimulated *Glossina palpalis gambiensis* flies

**DOI:** 10.3389/fmicb.2015.00444

**Published:** 2015-05-12

**Authors:** Anne Geiger, Illiassou Hamidou Soumana, Bernadette Tchicaya, Valérie Rofidal, Mathilde Decourcelle, Véronique Santoni, Sonia Hem

**Affiliations:** ^1^UMR 177, Institut de Recherche pour le Développement-CIRAD, CIRAD TA A-17/GMontpellier, France; ^2^Plateforme de Spectrométrie de Masse Protéomique – MSPP, Biochimie et Physiologie Moléculaire des Plantes - UMR 5004 Centre National de la Recherche Scientifique/UMR 0386 INRA/Montpellier SupAgro/Université Montpellier IIMontpellier, France

**Keywords:** sleeping sickness, tsetse-bacteria-trypanosomes, tripartite interactions, trypanosome-associated global changes, label-free quantification

## Abstract

The unicellular pathogenic protozoan *Trypanosoma brucei gambiense* is responsible for the chronic form of sleeping sickness. This vector-borne disease is transmitted to humans by the tsetse fly of the group *Glossina palpalis*, including the subspecies *G. p. gambiensis*, in which the parasite completes its developmental cycle. Sleeping sickness control strategies can therefore target either the human host or the fly vector. Indeed, suppression of one step in the parasite developmental cycle could abolish parasite transmission to humans, with consequences on the spreading of the disease. In order to develop this type of approach, we have identified, at the proteome level, events resulting from the tripartite interaction between the tsetse fly *G. p. gambiensis*, its microbiome, and the trypanosome. Proteomes were analyzed from four biological replicates of midguts from flies sampled 3 days post-feeding on either a trypanosome-infected (stimulated flies) or a non-infected (non-stimulated flies) bloodmeal. Over 500 proteins were identified in the midguts of flies from both feeding groups, 13 of which were shown to be differentially expressed in trypanosome-stimulated vs. non-stimulated flies. Functional annotation revealed that several of these proteins have important functions that could be involved in modulating the fly infection process by trypanosomes (and thus fly vector competence), including anti-oxidant and anti-apoptotic, cellular detoxifying, trypanosome agglutination, and immune stimulating or depressive effects. The results show a strong potential for diminishing or even disrupting fly vector competence, and their application holds great promise for improving the control of sleeping sickness.

## Introduction

Sleeping sickness in humans, or Human African Trypanosomiasis (HAT), is caused by two types of pathogenic protozoa. *Trypanosoma brucei gambiense* (Tbg), responsible for the chronic form of HAT, is transmitted to humans by the tsetse fly vector of the *Glossina palpalis* group (Hoare, 1972; Kazadi, 2000; Truc et al., 2011). This form of HAT is endemic in 24 African countries. In contrast, *T. b. rhodesiense* is transmitted by the vector of the *G. morsitans* group (Aksoy et al., 2013), and is responsible for the acute form of HAT in 13 East African countries (Welburn et al., 2009). Both forms of the disease represent a heavy burden to populations living within HAT risk areas; in parallel, *T. b. brucei, T. congolense*, and *T. vivax* cause the animal form of trypanosomiasis (AAT) and are responsible for dramatic losses to African livestock (Shaw et al., 2013).

Because sleeping sickness is a vector-borne disease, its control strategies can target either the human host (e.g., preventive and/or curative approaches) or the vector. Several anti-vector strategies are possible including chemicals, or the use of sterile males to eradicate tsetse fly populations (Abd-Alla et al., 2013). In addition, an approach that exploits the characteristics of trypanosome development within its vector could diminish the ability of tsetse flies to transmit the parasite, by reducing or even suppressing their vector competence.

*Glossina palpalis gambiensis* (Gpg), a strictly hematophagous fly, becomes infected while feeding on a Tbg-infected host (human or animal). After its ingestion, the trypanosome must achieve its developmental cycle within the fly and undergo several maturation steps from its procyclic into its metacyclic form. Since the latter is the only form that is infectious for mammals, the trypanosome must reach this stage before its transmission in a bloodmeal (Vickerman et al., 1988; Maudlin and Welburn, 1994). This suggests that suppressing one step in the parasite developmental cycle should interrupt parasite transmission to mammals and consequently the spreading of the disease.

The first and most crucial step in the trypanosome developmental cycle is its establishment in the fly's midgut. While some flies within a population are susceptible to trypanosome infection, most are naturally able to eliminate the ingested trypanosomes (i.e., to self-cure) and are thus resistant (refractory) to infection. This elimination process occurs after the bloodstream form of the ingested trypanosomes has differentiated into the procyclic form (at 24–72 h following parasite ingestion), and lasts for approximately 3 days (Van den Abbeele et al., 1999; Aksoy et al., 2003; Gibson and Bailey, 2003). This indicates that a molecular crosstalk occurs at an early step of infection, resulting in the induction of factors that favor either the fly's susceptibility or refractoriness. The fly and the invading parasite are not the only partners in this crosstalk, which also includes (at least) the *Glossina* secondary symbiont *Sodalis glossinidius*; this species was previously demonstrated to promote fly infection (Geiger et al., 2007; Farikou et al., 2010). Furthermore, the transcriptomes of *S. glossinidius* and *Wigglesworthia glossinidia*, the tsetse fly obligate symbiont, were shown to be modified following fly infection by the trypanosome (Hamidou Soumana et al., 2014a,b). Finally, it is plausible that the whole microbiome of the tsetse fly (Geiger et al., 2013) may be involved in modulating the fly's global response to trypanosome invasion, and consequently its vector competence.

Numerous reports in the literature have emphasized the complex nature of the mechanisms involved in tsetse vector competence. Recently, it was shown that antioxidants could increase fly susceptibility (MacLeod et al., 2007). In addition, the obligate tsetse symbiont *Wigglesworthia* may indirectly constrain pathogen development by affecting the host peptidoglycan recognition protein PGRP-LB (Wang et al., 2009; Weiss et al., 2013). Furthermore, tsetse EP (glutamic acid-proline) proteins could offer protection from trypanosome establishment (Haines et al., 2010). These proteins are strongly up-regulated after challenging tsetse flies with Gram-negative bacteria (Haines et al., 2005), suggesting a possible role in the insect immune response. Likewise, injecting *Escherichia coli* to up-regulate the immune response leads to a significant reduction in trypanosome prevalence (Haines et al., 2005; Hu and Aksoy, 2006). Finally, the procyclic form of different trypanosome species has been shown to secrete different proteins *in vitro* (Atyame Nten et al., 2010).

By contrast, few reports in the literature have focused on the early trypanosome invasion step. In an effort to provide an alternative disease control strategy, we aimed to identify, at the proteome level, events resulting from the interaction between the tsetse fly, its microbiome, and the trypanosome. These experiments were performed on the midguts of insectary-reared Gpg flies. As previously shown, these flies harbor the obligate symbiont *W. glossinidia* and the secondary symbiont *S. glossinidius* (Hamidou Soumana et al., 2013). We first analyzed the proteomes of midguts from flies that received either a trypanosome-infected or non-infected bloodmeal. Subsequently, these proteomes were compared in order to identify the proteins that are differentially produced under the two conditions. Finally, the function of these proteins and their potential role in tsetse infection is discussed.

## Materials and methods

### Ethical statement

All experiments on animals were conducted according to internationally recognized guidelines. Experimental protocols were approved by the Ethics Committee on Animal Experiments, and the Veterinary Department of the Centre International de Recherche Agronomique pour le Développement (CIRAD), Montpellier, France.

### Tsetse flies and trypanosomes

Gpg flies originated from colonies (CIRAD insectary, Montpellier) fed on rabbits. Strain *T. b. gambiense* S7/2/2 was isolated in 2002 by rodent inoculation with blood from HAT patients diagnosed in the sleeping sickness focus of Bonon, Ivory Coast (Ravel et al., 2006). Cryostabilates of S7/2/2 were thawed and injected intraperitoneally into BALB/c mice. To monitor murine infections, tail blood samples were examined by phase contrast microscopy until the parasitemia count reached 16-64 × 10^6^ parasites/ml (i.e., 27–50% stumpy form).

As the susceptibility of the flies to trypanosome infection varies with the fly's age (Walshe et al., 2011), the age of the teneral female *G. p. gambiensis* flies under experiment were similar, and around 30 h post eclosion. The flies were fed on the blood of either a trypanosome-infected or non-infected mice. Several mice were needed to feed all the flies. After feeding, the flies fed on non-infected mice were grouped altogether, the non-gorged flies were removed, and finally among the remaining flies, 28 randomly chosen individuals were dissected; the midgut of each fly was kept in separate dry tube at –80°C until protein extraction. Before protein extraction, four biological replicates, each of seven randomly selected midguts were constituted. These replicates were called “NS” (midguts from non-stimulated flies) replicates. The four “S” replicates (midguts from stimulated flies = from flies fed on infected mice) were processed similarly.

### Total proteome preparation

Midguts were ground with pistons, and proteins were extracted in Laemmli's buffer (Laemmli, 1970) prepared with cOmplete Mini Protease Inhibitor (Roche) and Pefabloc SC (Roche). The extracts were then sonicated for 30 min. Total soluble protein fractions were recovered by centrifugation at 14,000 g for 10 min at room temperature, and protein concentrations were measured using a 2D Quant kit (GE Healthcare).

### One-dimensional electrophoretic analysis

**Figure 1:**
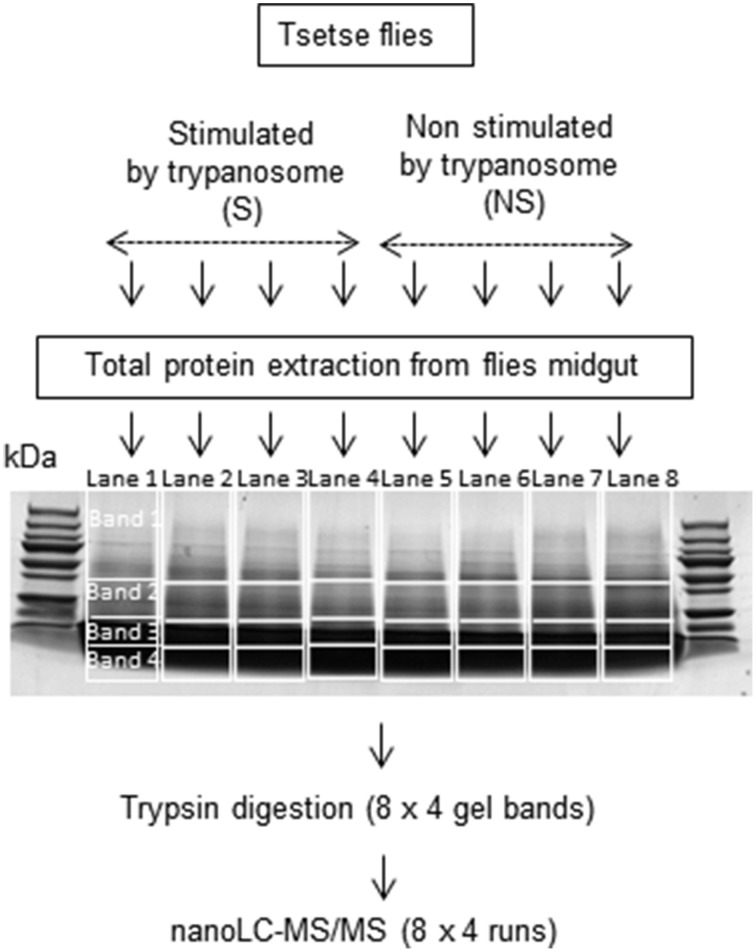
**Fractionation of proteins by 1D SDS-PAGE**. Lanes 1 to 4: midgut samples from flies fed on an infected bloodmeal. Lanes 5 to 8: midgut samples from flies fed on a non-infected bloodmeal.

Twenty microgram of proteins from the different samples (four biological replicates per condition) were heated at 90°C for 5 min and centrifuged for 5 min at 14,000 g prior to separation by one-dimensional SDS-PAGE. Proteins were applied to eight wells and separated on 10 × 7 cm Tris/glycine PAGE gels (12% acrylamide Mini-PROTEAN TGX precast gels, Biorad). After a short 1.5 cm migration (to fractionate the sample) into the resolving gel, gels were fixed and the proteins were visualized with Coomassie brilliant blue R-250. Gel images were obtained with a high-resolution scanner (Amersham Biosciences) (Figure 1). Each lane was sliced horizontally into four bands and washed with 1 mL of water followed by 1 mL of 25 mM NH_4_HCO_3_. Destaining was performed twice in the presence of 1 mL of 50% acetonitrile in 25 mM NH_4_HCO_3_. Gel bands were dehydrated twice in 1 mL of 100% CH_3_CN and finally dried at room temperature. Destaining was followed by reducing disulfide bridges with 250 μl of 10 mM DTT at 56°C for 45 min; the supernatant was then removed and cysteine groups were alkylated with 250 μl of 55 mM iodoacetamide for 30 min on a vortex in the dark. Gel bands were washed twice with 1 ml of 50% acetonitrile in 25 mM NH_4_HCO_3_. Bands were subsequently dehydrated in 1 mL of 100% CH_3_CN and finally dried at room temperature. Twenty microliters of a trypsin solution (Sequencing Grade Modified Trypsin, Promega; Madison, USA) were added to each gel piece at a concentration of 0.0125 μg/μL in 25 mM NH_4_HCO_3_ and maintained on ice for 15 min. Twenty microliters of 25 mM NH_4_HCO_3_ were added, and the samples were maintained another 15 min at room temperature. Protein digestion was performed overnight at 37°C and stopped by addition of 100 μl of 2% formic acid with sonication in an ultrasonic bath for 10 min. Supernatants containing trypsic peptides were transferred into a 0.5-mL glass insert. The remaining trypsic peptides were extracted twice from bands by addition of 100 μL of 80% acetonitrile in 2% formic acid. Extracted peptides were pooled in glass inserts and then dried under vacuum. Peptides were then resuspended in 10 μl of a 2% formic acid solution before LC-MS/MS analysis.

### Nano LC–MS/MS

Protein digests were analyzed by a Q-TOF mass spectrometer (Maxis Impact, Bruker Daltonik GmbH; Bremen, Germany) using a CaptiveSpray source and interfaced with a nano-HPLC U3000 system (Thermo Scientific; Waltham, USA). Samples were concentrated on a pre-column (Thermo Scientific, C18 PepMap100, 300 μm × 5 mm, 5 μm, 100 A) at a flow rate of 20 μL/min using 0.1% formic acid. After pre-concentration, peptides were separated on a reversed-phase capillary column (Thermo Scientific, C18 PepMap100, 75 μm × 250 mm, 3 μm, 100 A) at a flow rate of 0.3 μL/min using a two-step gradient (2–25% acetonitrile for 97 min, followed by 25–42% acetonitrile for 10 min) and eluted directly into the mass spectrometer. The mass range was measured from 120 to 2800 m/z. Twenty major ions were selected for fragmenting and were then excluded during 0.2 min.

### Protein identification with reference to databases

MS/MS raw data were analyzed using the Data Analysis software (Bruker Daltonik GmbH; Bremen, Germany) to generate the peak lists. The resulting mgf (Mascot Generic Format) files were then searched against a home-built database (555,275 entries) made of compiled *Drosophila*, mouse, *Glossina, Sodalis, Wigglesworthia*, and *Trypanosoma* protein databases from UniProtKb (2013-07-12).

This local database was queried using the Mascot search engine v.2.2.07 (Matrix Science, http://www.matrixscience.com) and included: all entries for taxonomy; trypsin as an enzyme; one missed cleavage allowed; and carbamidomethylation of cysteine as a fixed modification. N-terminal acetylation, deamidation of asparagines and glutamines, methionine oxidation, and N-terminal pyroglutamylation of glutamic acid and glutamine were also included as variable modifications. Mass tolerance was set at 10 ppm for full MS scans, and 0.05 Da for fragment ions. Protein identification was validated once proteins contained at least one unique peptide (i.e., not shared with another accession in the used database) with a *p*-value < 0.05.

### Label-free quantification

The IDEAL-Q software (ID-based Elution time Alignment by Linear regression Quantification; Tsou et al., 2010) was combined with an in-house program known as “IDEAL-DB” to generate the quantification data. For each fraction (SDS bands), Maxis Impact raw data were converted into the mzXML format by CompassXport (Data Analysis, Bruker Daltonik). The mzXML files and the Mascot search results for peptide and protein identification were used as inputs for the quantitative tool. Alignment of elution times was performed by the IDEAL-Q algorithm based on the list of peptides identified by Mascot for all analyses. This step allowed fixing potential shifts in retention time between LC-MS runs. Moreover, peptide ions that were not initially identified by Mascot (due to low abundance and/or mass spectrometer limitations) were recovered by this algorithm, and the corresponding area was extracted. To ensure correct assignment of the detected peaks, peptides were validated by taking into account the following criteria: a signal-to-noise ratio above 30; a correct charge state; and an experimental isotopic pattern corresponding to the theoretical pattern (Tsou et al., 2010). The abundance of the parent peptide was calculated according to the area under the curve from the extracted ion chromatograms (XIC).

Peptide characteristics generated by IDEAL-Q (i.e., peptide sequence, charge state, elution time, and area) were used as inputs for IDEAL-DB. The latter software was used to normalize the peptide areas, by dividing the area of each quantified peptide by the sum of the areas of all quantified peptides within its LC-MS run. Since a peptide can be present in different SDS bands, the abundance of a peptide in one sample (corresponding to one biological replicate, i.e., one SDS-PAGE gel lane) was calculated by summing the normalized areas of the peptide in each lane (Gautier et al., 2012). The peptide abundance in one biological condition was then determined by the average of its area values in the different biological replicates. At the protein level, only unique and unmodified specific peptides present in at least three of the four biological replicates were used to calculate a protein quantitative ratio. Modified peptides were considered, provided that the unmodified counterpart was also quantified. Protein abundance was calculated by summing peptide abundances in each experimental condition. The protein abundance ratio and the corresponding Student's *t*-test were calculated between the two experimental conditions (stimulated vs. non-stimulated). Proteins with a significant quantitative ratio (*p* < 0.05) above 1.2 or below 0.8 were considered.

### Functional annotation

The Mass Spectrometry Data Analysis site (https://msda.unistra.fr/) was used to obtain the Gene Ontology (GO) annotation of proteins.

## Results

### Quantification of proteins in tsetse flies fed on either an infected or non-infected bloodmeal

We anticipated an impaired identification of low abundance proteins due to the complexity of unfractionated lysates in the tsetse midgut. Therefore, proteins were fractionated by one-dimensional SDS-PAGE (Figure 1) to improve analysis and allow identification of even minor proteins by LC–MS/MS analysis. The results (Supplementary Tables S1–S8) indicate that proteins of very low abundance (0.01 Protein Abundance Index or PAI) could be identified in addition to others of very high abundance (PAI > 200,000). However, it cannot be excluded that proteins with a PAI < 0.01 could be present but undetectable in the midgut extracts. Indeed, only several *Wigglesworthia, Sodalis* and trypanosome proteins were revealed. Symbiont proteins were observed in the midguts of both trypanosome-stimulated and non-stimulated flies, whereas trypanosome proteins were only detected in stimulated flies. Table 1 presents the identity of several such proteins and their distribution among the different repeats. A limited number of proteins were identified as belonging to bacteria within the genera *Providentia* and *Acinetobacter*. The presence of these bacteria in the midgut of tsetse flies was reported previously in flies collected within HAT foci (Geiger et al., 2009, 2011; Lindh and Lehane, 2011). However, their presence in laboratory colonies was not yet reported, possibly because the culture method previously used to isolate the bacteria was not enough sensitive.

**Table 1:** **Example of protein abundance variability between the different biological replicates**.

**Accession**	**Description**	**Protein Abundance Index (emPAI)**							
		**Replicates from stimulated flies (S)**				**Replicates from non-stimulated flies (NS)**			
		**S-A**	**S-B**	**S-C**	**S-D**	**NS-A**	**NS-B**	**NS-C**	**NS-D**
tr|D3TLL9|D3TLL9_GLOMM	Fatty acid-binding protein FABP OS= Gmm	10.43	24.76	30.56	16.16	16.16	5.22	24.76	46.37
tr|Q8IS91|Q8IS91_GLOFF	Phosphotrypsin OS=G. fuscipes fuscipes	8.42	5.84	5.84	6.61	5.14	3.46	5.14	9.48
tr|D3TLW4|D3TLW4_GLOMM	Midgut trypsin OS=Gmm PE=2 SV=1	3.59	2.75	2.75	3.15	2.06	1.76	2.38	4.08
tr|D3TRY2|D3TRY2_GLOMM	Porin OS=Glossina morsitans morsitan	3.23	6.85	4.20	4.24	3.69	3.23	7.70	9.70
tr|D3TRW4|D3TRW4_GLOMM	ATP synthase subunit beta OS=Gmm	3.09	6.35	5.54	3.33	3.38	3.87	5.93	2.59
tr|D3TQR2|D3TQR2_GLOMM	Glutamate dehydrogenase OS=Gmm	2.87	2.87	3.30	2.68	2.68	2.31	3.53	5.87
tr|D3TR42|D3TR42_GLOMM	ATP synthase subunit alpha OS=Gmm	2.83	5.21	4.89	3.50	4.29	3.26	5.55	6.70
tr|D3TNQ0|D3TNQ0_GLOMM	ADP/ATP translocase OS=Gmm PE=2 SV=1	2.81	5.15	5.15	4.59	3.20	5.15	6.45	9.92
tr|D3TRU0|D3TRU0_GLOMM	Glyceraldehyde-3-phosphate DH OS=Gmm	2.77	3.91	4.37	2,16	2.45	1.42	3.91	5.99
tr|D3TPC2|D3TPC2_GLOMM	Hypothetical conserved protein OS=Gmm	2.46	3.13	1.90	2.46	1.03	1.03	1.43	2.46
tr|D3TNY9|D3TNY9_GLOMM	Putative transl. Initiat. Inhibitor Gmm	2.44	3.23	5.38	3.23	1.80	0.85	2.44	6.84
tr|D3TS28|D3TS28_GLOMM	Differentiation-related protein 1 protein Gmm	2.07	2.33	1.62	1.84	2.07	2.07	3.97	3.23
tr|D3TPN5|D3TPN5_GLOMM	Arginine kinase OS=Gmm PE=2 SV=1	2.01	2.25	2.25	1.57	1.38	1.09	2.25	3.46
tr|D3TQ00|D3TQ00_GLOMM	Myosin heavy chain OS=Gmm PE=2 SV=1	1.91	2.13	1.91	1.25	1.51	1.70	3.35	5.53
tr|D3TP69|D3TP69_GLOMM	60s acidic ribosomal protein P1 OS=Gmm	1.89	2.77	1.89	1.22	0.70	1.89	1.89	1.89
tr|Q694A5|Q694A5_GLOMM	Putative thioredoxin peroxidase 1 OS=Gmm	1.69	1.33	1.03	1.69	1.33	1.33	1.33	2.57
tr|D3TLD9|D3TLD9_GLOMM	Putative aminopeptidase OS=Gmm	1.65	2.54	1.65	1.51	1.11	1.37	1.81	1.98
tr|D3TRM9|D3TRM9_GLOMM	Superoxide dismutase [Cu-Zn] OS=Gm	1.60	1.15	0.77	0.47	0.47	0.77	1.15	1.15
tr|D3TL85|D3TL85_GLOMM	Ribosomal protein S25 (Fragment) OS=m	1.53	1,01	1.01	1.53	0.59	1.01	1.53	1.53
tr|D3TQT6|D3TQT6_GLOMM	Glycerol 3 phosphate dehydrogenase (Gmm)	1.41	1.83	1.83	1.61	1.41	1.83	2.32	3.22
tr|D3TKT4|D3TKT4_GLOMM	Profilin (Fragment) OS=Gmm PE=2 SV=1	1.41	4.80	6.23	3.66	2.74	2.74	6.23	8.01
tr|D3TNV2|D3TNV2_GLOMM	Enolase OS=Glossina morsitans morsitans	1.38	1.54	1.72	1.72	1.23	1.38	1.91	2.11
tr|D3TSL2|D3TSL2_GLOMM	Flavin reductase OS=Gmm PE=2 SV=1	1.28	2.00	2.00	1.28	1.28	0.99	2.00	3.53
tr|D3TNV8|D3TNV8_GLOMM	Elongation factor 1-alpha OS=Gmm	1.13	1.27	1.74	1.41	1.41	1.74	2.11	1.92
tr|D3TRW6|D3TRW6_GLOMM	Ribosomal protein L30 OS=Gmm	1.08	3.31	1.65	1.65	1.65	1.65	7.96	4.50

To identify the molecular components found in midguts from flies after an infected (“stimulated”) or non-infected (“non-stimulated”) bloodmeal, we considered proteins with peptide scores greater than the identity threshold (*p* < 0.05). MS/MS analysis identified more than 500 proteins in our database from each of the eight tsetse midgut samples (four non-stimulated and four trypanosome-stimulated replicates). The complete list of proteins identified from each sample is provided in Supplementary Tables S1–S8. Table 1 presents a list of 25 among the most abundant proteins identified in the “S-A” replicate (corresponding to Supplementary Table S1) with reference to *Glossina* data base. The abundance index of the corresponding proteins found in the seven other replicates were then provided. This table is provided to get a rough/visual evaluation of the abundance variability occurring between the “S” and “NS” replicates.

The quantitative differences in protein abundance between the overall proteins from stimulated and non-stimulated midguts were examined by a label-free quantitative proteomic approach.

Proteomes from stimulated and non-stimulated flies (comprising four biological replicates each) were analyzed to examine the possible occurrence of biological variations across data sets prior to protein quantification. The fold-change in protein quantity was calculated as the ratio of protein abundance (see the Materials and Methods section) between midguts from stimulated and non-stimulated flies. Despite the large variability in protein abundance, as shown for example in Table 1, 13 proteins were determined to exhibit significant abundance differences in flies fed an infected bloodmeal. All up- and down-regulated proteins (in stimulated vs. non-stimulated flies) and their corresponding quantified peptides are presented in Table 2 and Supporting Table S9. Three of the 13 proteins were up-regulated, whereas 10 were down-regulated following trypanosome stimulation. Significant proteins that were differentially expressed and identified in tsetse flies fed an infected bloodmeal (vs. those fed a non-infected bloodmeal) correspond to insect proteins.

**Table 2:** **Proteins significantly up- or down-regulated following trypanosome stimulation**.

**Protein description**	**UniProtKB Accession**	**Ratio**	***p*-value**	**Number of peptides quantified**	**Peptide sequence**
Isocitrate dehydrogenase (NAD+)	B4GFZ8 (a) Q0QHL1 (b) Q295M2 (c)	2.14	0.033	1	TDIPSAQYGGR
GK22983 (GST family)	B4NN02 (d)	1.91	0.034	1	LHFESGVIFEGALR
Lectizyme	Q8MUG0 (b)	1.56	0.009	1	VNLPTGKYESTGK
Eukaryotic translation initiation factor 3	D3TMN6 (b)	0.18	0.027	1	ELQISEDEVEPFVIEVLK
Putative membrane protein	D3TSM2 (b)	0.4	0.026	1	NTDTQDELEEVQSDLR
GI12924	B4KZE6 (e)	0.42	0.033	1	QPLISR
Acyl-coenzyme A oxidase	D3TSF3 (b)	0.51	0.023	1	AVCSADAASGVEVCR
Cytochrome c oxidase	D3TRY9 (b)	0.53	0.041	1	ASFCQTFAEIQAPTGEFK
Glycerol 3 phosphate	D3TQT6 (b)	0.59	0.031	5	IVGANCAALPEFEDR
dehydrogenase					VVVVQDSDAVEICGALK LTEIINTSHENVK TLRDLFQSENFR DLFQSENFR
4-hydroxybutyrate coenzyme A transferase	D3TLS8 (b)	0.64	0.047	1	SGDTVFTSGAAATPK
Eukaryotic translation initiation factor 3	D3TRV5 (b)	0.66	0.007	1	TVDATTADQSPILR
1-pyrroline-5-carboxylate dehydrogenase 2	Q0QHK6 (b)	0.71	0.020	12	DIDKANYIVQGLR EEIFGPVQQIIR TIPMDGDFFAYTR TIPM[Oxidation(M)]DGDFFAYTR ILQLIDSGKQQGAK (di +tri) VAFTGSTEVGK AGKEDVDLAVQAAR (di+ tri) IAREEIFGPVQQIIR LIQQASGNTNLKR (di + tri) LIQQASGNTNLK TFPSINPTTEK ANYIVQGLR
GDP-mannose pyrophosphorylase	D3TND8 (b)	0.73	0.020	1	LHSGPGIVGNVLVDPSAK

*a, Drosophila persimilis; b, Glossina sp.; c, Drosophila pseudoobscura pseudoobscura; d, Drosophila willistoni; e, Drosophila mojavensis; ratio, ratio (stimulated flies/non-stimulated flies); p-value, Student's t-test*.

### Functional annotation of proteins identified by LC-MS/MS

**Figure 2:**
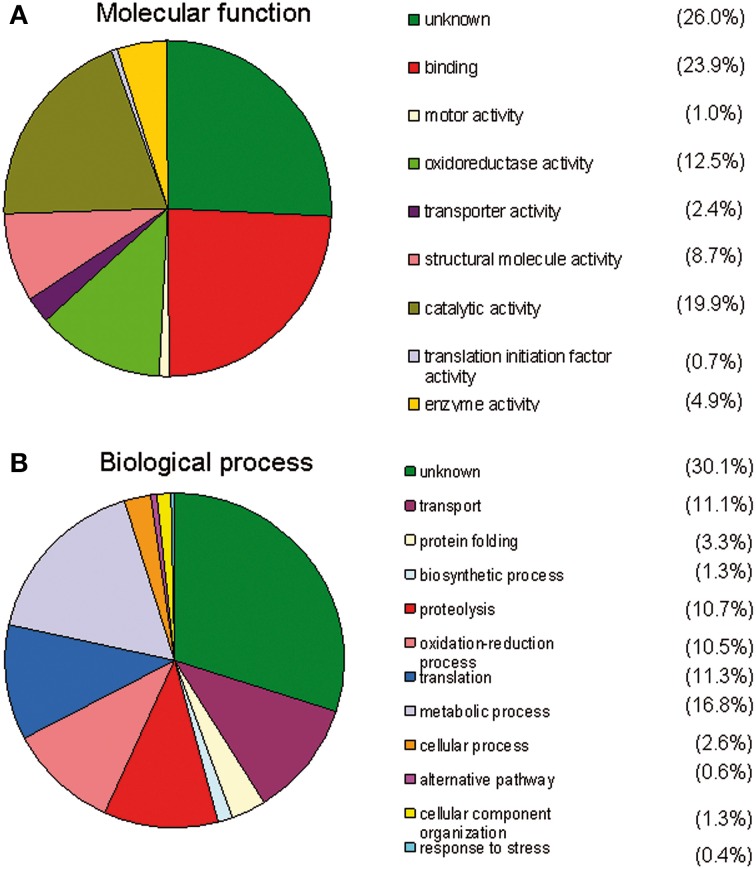
**Gene ontology analysis using the MSDA database**. The different groups are ranked by size. **(A)** Analysis of the molecular functions of the identified midgut proteins. Many of the proteins display a binding function, oxidoreductase activity, or have unknown functions. **(B)** Analysis of the biological processes of the identified midgut proteins A number of proteins are involved in metabolic processes, proteolysis, oxidation-reduction processes or have unknown functions.

Overall data from gene ontology analysis suggest that most of the identified proteins in each of the midgut samples (from stimulated and non-stimulated flies) are involved in critical events such as DNA replication, translation initiation or elongation, binding, proteolysis, protein transport, oxidation reduction, response to oxidative stress, metabolic processes (involving sugars or lipids), biosynthetic processes, and catalytic activity. Additional information on their molecular functions was obtained by a GO analysis using the MSDA database. Molecular function analysis revealed that most of the known proteins are involved in binding (23.9%), catalytic activity (19.9%), and oxidoreduction activity (12.5%) (Figure 2A). Furthermore, 70% of the proteins were found to be involved in 11 different biological processes, the most quantitatively important being: metabolic processes (16.8%), proteolysis (10.7%), oxidoreduction (10.5%), transport (11.1%), and translation processes (11.3%) (Figure 2B).

### Up- and down-regulated proteins

The three up-regulated proteins include isocitrate dehydrogenase, glutathione S-transferase and lectizyme (Table 2), as identified in UniprotKB. The first two proteins are involved in the tricarboxylic acid cycle, and the third protein plays a role in proteolysis.

Proteins that were down-regulated following trypanosome stimulation were mostly involved in protein synthesis, fatty acid oxidation, nucleotidyl transferase activity, multicellular organism development, oxidoreductase activity, and carbohydrate metabolic processes (Table 2). Among these proteins, we identified a midgut initiation factor, a cytochrome c oxidase, an oxidoreductase, an acyl-coenzyme A oxidase, a GDP-mannose pyrophosphorylase, a transferase, a protein involved in gluconeogenesis, a putative membrane protein, and a protein involved in development.

## Discussion

### General comments

Even under ideal artificial infection conditions in the laboratory, frequently only 15–20% of tsetse flies fed on a trypanosome-infected bloodmeal become infected (Ravel et al., 2006). This number is significantly reduced in field populations, where infection prevalence seldom exceeds 10% (Moloo et al., 1986; Dukes et al., 1989; Maudlin and Welburn, 1994). This indicates that most tsetse flies within a population are refractory to trypanosome infection. Many factors participate in the success or failure of the infection and maturation processes (Jordan, 1986; Maudlin and Welburn, 1994), in particular fly immunity (Welburn and Maudlin, 1999; Hao et al., 2001).

The first line of defense in the tsetse midgut is the peritrophic matrix, which forms a barrier surrounding the entire digestive tract (Moloo et al., 1970; Miller and Lehane, 1990; Miller, 1991; Lehane, 1997). The second obstacle to infection is the attrition process that parasites undergo in the midgut approximately 3 days post-infected bloodmeal (Gibson and Bailey, 2003). It has also been suggested that midgut lectins may be involved in tsetse fly defense. In fact, feeding on specific sugars (that act as lectins inhibitors) considerably increases trypanosome midgut infection rates (Maudlin and Welburn, 1987). In addition to lectins and agglutinins, reactive intermediates may participate in mediating refractoriness (MacLeod et al., 2007).

In this context, the early events following the uptake of an infected bloodmeal (and that begin with the trypanosome invasion of the tsetse fly midgut) are of crucial importance. Our experiments on flies 3 days after ingestion of an infected or non-infected bloodmeal had several objectives: (i) to characterize the global proteome of the tsetse midgut at an early step of the vector-parasite interaction; (ii) to determine the proteins that are differentially produced (either up- or down-regulated); (iii) to identify protein functions involved in this host-parasite dialog; and (iv) to determine proteins or biosynthetic pathways that may be valuable to a potential anti-vector strategy.

Over 500 proteins were identified in each sample. In previous study by Haddow et al. (2005), in which the midgut proteome of trypanosome-susceptible *Glossina morsitans morsitans* (Salmon mutant) was compared to those of wild *G. m. morsitans* succeed to identify 207 proteins using isotope coded affinity tag (ICAT). We expected a high number of proteins, since the midgut proteins included the global pool of enzymes involved in diverse metabolic pathways and all other soluble proteins. We also expected the presence of exogenous proteins, including from mice (i.e., the bloodmeal source) as well as from trypanosomes (in the midguts of trypanosome-stimulated flies). In fact, a number of proteins showing a very high abundance index were identified, which relate to mice. This list of proteins includes alpha- and beta-globins, and hemoglobin (see Supplementary Tables S1–S8). These results indicate that by day 3 post-bloodmeal the flies had not yet digested all of the ingested mouse proteins. It is possible that high concentrations of these blood proteins may impair the detection of low-abundance proteins. However, delaying the experiment to day 5 or even later would result in the impaired detection of early differential biosynthetic events. Recently, Rose et al. (2014) reported the identification of 300 proteins as components of the teneral *G. m. morsitans* peritrophic matrix. Almost all the most abundant proteins identified in their study were also identified in ours, except for example, peritrophin-like protein, proventriculin or chitinase precursor, that may be masked by the over-abundance of some proteins corresponding probably to mice proteins ingested with the blood meal and not yet degraded.

Another aspect has to be considered as within a population of flies, 15–20% only will become infected even under optimized experimental infection. This means that 80% of the flies are expected to be refractory to trypanosome infection, and, thus, that most of the proteins extracted from the “NS” flies will be from refractory flies and may affect the identification of proteins involved in susceptibility. In fact, two possibilities could be considered. Either the differences between infected flies (all of them are susceptible flies) and “control” flies will be enhanced. But one may also consider that the protein signature of non-infected flies would be similar, whether the flies are genetically programmed for susceptibility or refractoriness. This would mean, that proteins involved in refractoriness are not constitutive, and that their biosynthesis by refractory flies would be induced by the trypanosomes after being ingested by these flies (and thereafter eliminated by them). So the question remains open for further investigations.

The possibility to discriminate susceptible from refractory flies using the *Sodalis* genotyping approach could also be considered (Geiger et al., 2007); in the present study this approach could not be performed as the extraction process was focused on protein, not on DNA analysis.

Finally, a question may also be considered regarding the location of the identified proteins: will they be and remain strictly intracellular or may they be secreted in the extracellular compartment where they will get the possibility to impact the incoming trypanosomes? Thus, further studies on presence/absence of signal peptides should be done as previously (Geiger et al., 2010).

From our study, very few trypanosome proteins were detected in stimulated flies (i.e., in flies that ingested a trypanosome-infected bloodmeal; Table 3). Almost none of the previously characterized proteins from the trypanosome proteome or secretome (Atyame Nten et al., 2010; Geiger et al., 2010) were identified in the midguts of stimulated flies. The absence of these proteins is most probably due to the attrition phenomenon that occurs after trypanosome ingestion, which results in a drastic decrease of the ingested trypanosome population within the fly's midgut (Gibson and Bailey, 2003). This may create a situation in which the corresponding trypanosome proteins are too low for detection. Similarly, only a limited number of proteins could be identified from *Wigglesworthia* and *Sodalis*, respectively the primary (obligate) and secondary symbionts of the tsetse fly (Table 3). For *Wigglesworthia*, this includes chaperons and chaperonins, thiamine biosynthesis protein, elongation factor; the *Sodalis* proteins include chaperonins, membrane proteins, elongation factors and a “hypothetical phage protein.” Previous studies of the *Sodalis* and *Wigglesworthia* transcriptomes revealed high differential expression of genes in both symbionts according to the status (i.e., trypanosome-infected, non-infected or self-cured) of their host flies (Hamidou Soumana et al., 2014a,b). Interestingly, our detection of a hypothetical phage protein recollects the presence of a previously reported prophage in the *Sodalis* genome (Hamidou Soumana et al., 2014a).

**Table 3:** **A selection of bacterial and trypanosomal proteins identified in the biological repeats of trypanosome-stimulated and non-stimulated *Glossina palpalis gambiensis* midgut extracts**.

**Accession**	**Description**	**Protein abundance index**							
		**NS-A (ST5)**	**NS-B (ST6)**	**NS-C (ST7)**	**NS-D (ST8)**	**S-A (ST1)**	**S-B (ST2)**	**S-C (ST3)**	**S-D (ST4)**
***WIGGLESWORTHIA* PROTEINS**									
tr|H6Q558|H6Q558_WIGGL	60 kDa chaperonin (GN=groL PE=3 SV=1)	0.74	1.05	2.38	3.46	0.94	1.56	1.05	0.84
tr|H6Q518|H6Q518_WIGGL	Chaperone protein DnaK	0.20		0.25	0.31	0.20			0.20
sp|Q8D2Q5|DNAK_WIGBR	Chaperone protein DnaK		0.20				0.20	0.20	
tr|H6Q557|H6Q557_WIGGL	10 kDa chaperonin (GN=groS PE=3 SV=1)		3.19		8.89		6.43	6.43	
tr|Q8D267|Q8D267_WIGBR	AhpC protein	0.15		0.15	0.15	0.15	0.15	0.15	0.15
tr|B7U9A5|B7U9A5_WIGGL	Thiamine biosynthesis protein ThiC (Fragment)	0.07	0.15	0.32		0.15	0.15		
sp|Q8D240|EFTU_WIGBR	Elongation factor Tu	0.08	0.16	0.24	0.24	0.16	0.16	0.24	
tr|H6Q4M2|H6Q4M2_WIGGL	Elongation factor Tu			0.16	0.16			0.16	
***SODALIS* PROTEINS**									
sp|Q2NW95|CH10_SODGM	10 kDa chaperonin (GN=groS PE=3 SV=1)	2.15	3.19		4.58	4.58		6.43	2.15
tr|Q2NU70|Q2NU70_SODGM	Outer membrane protein A			0.09		0.09	0.09		0.09
tr|Q2NSM2|Q2NSM2_SODGM	Outer membrane protein	0.08	0.17	0.26	0.08		0.17	0.08	
tr|Q2NSB6|Q2NSB6_SODGM	Hypothetical phage protein		0.06	0.06		0.06	0.12		0.06
sp|Q2NQL7|EFTU_SODGM	Elongation factor Tu		0.08	0.08	0.08		0.16	0.08	
***TRYPANOSOMA* PROTEINS**									
tr|K2N4W8|K2N4W8_TRYCR	Mismatch repair protein MSH5, putative					0.04	0.04	0.04	0.04
tr|Q4DW89|Q4DW89_TRYCC	Lipophosphoglycan biosynthetic protein putative					0.04	0.04	0.04	0.04
tr|D0A9H8|D0A9H8_TRYB9	Calmodulin, putative (Fragment)					0.20	0.44	0.20	0.20
tr|B0M0I4|B0M0I4_TRYEV	Beta tubulin (Fragment)					0.15	0.15		0.15
tr|K2MW43|K2MW43_TRYCR	Ubiquitin-protein ligase, putative					0.01	0.01	0.01	
tr|C9ZM94|C9ZM94_TRYB9	Putative uncharacterized protein					0.03	0.08	0.03	0.03

*NS, Non-stimulated fly repeats; S, trypanosome-stimulated fly repeats; (ST), Corresponding Supplementary Table reference number*.

### Specific comments

The proteins were categorized into three groups based on their activity, function, and involved biological process (Figures 2A,B). The top results from the 27 categories of characterized protein activities and the “unknown” proteins group include: oxidoreductase, catalytic activity, structural proteins, peptidase, nucleotide binding, and ion binding.

Many proteins were involved in oxidoreduction mechanisms (Figure 2A) as expected, since hemes present in the bloodmeal in addition to trypanosomes may cause significant oxidative stress for flies (Hao et al., 2001; Lehane et al., 2003; Hu and Aksoy, 2006; MacLeod et al., 2007; Nayduch and Aksoy, 2007; Haines et al., 2010). Oxidative stress, characterized by the accumulation of reactive oxygen species, is known to have numerous detrimental effects on cells, including the induction of apoptotic cell death. Interestingly, a significant increase in H_2_O_2_ was observed in the presence of the bloodstream form of the parasite (Hao et al., 2003). This activity may be involved in trypanosome elimination during the first stages of the infection. Activation of other immune gene products involved in refractoriness may be due to H_2_O_2_ presence in the proventriculus (Hao et al., 2003). A similar role has been revealed for diptericin induction when NO is introduced into the hemolymph of normal *Drosophila* flies (Nappi et al., 2000). NO has also been shown to limit the development of malarial parasites in the mosquito *Anopheles stephensi* (Luckhart et al., 1998).

Twelve biological processes were also characterized, which were predominated by metabolic processes and unknown functions (Figure 2B). The proteins with the greatest potential were significantly differentially expressed (*p* = 0.05/Student's *t*-test) after fly stimulation by feeding on a trypanosome-infected bloodmeal. Twenty eight peptides corresponding to 13 proteins matched this criterion. Some of these proteins were down-regulated, whereas others were up-regulated in stimulated flies (vs. non-stimulated flies) (Table 2). Haddow et al. (2005) identify, previously, 17 midgut proteins that were up regulated and nine proteins down regulated in the trypanosome susceptible *G. m. morsitans* (Salmon mutant) as compared to normal (wild) *G. m. morsitans*. Very few of these proteins were shown to be differentially expressed in our experimental design. It may also be noted that none of the differentially expressed proteins we have evidenced (Table 2) are listed among the most abundant proteins from peritrophic matrix identified by Rose et al. (2014).

Several proteins that we have identified as overexpressed in stimulated flies include isocitrate dehydrogenase, glutathione S-transferase, and lectizyme. Former work carried out *in vitro* on H9c2 cardiomyocytes showed that isocitrate dehydrogenase is involved in antioxidant and anti-apoptotic mechanisms (Lee and Park, 2013). In addition, it is a key enzyme in the tricarboxylic acid (TCA) cycle, and could have an important impact on bacterial growth (Zhao et al., 2014). Glutathione S-transferases (GSTs) are a group of multifunctional enzymes that play a critical role in cellular detoxification. The contribution of mouse intestinal GST to the biochemical defense against *Trichinella spiralis* infection has previously been reported (Wojtkowiak-Giera et al., 2011). In the case of the trypanosome-tsetse fly interaction, isocitrate dehydrogenase and GST could be produced by flies to protect themselves in response to the presence of heme in the bloodmeal (in both non-stimulated and trypanosome-stimulated), or to the presence of trypanosomes in the bloodmeal upon entry into the midgut. Both enzymes display antioxidant effects and their over-expression, in trypanosome-stimulated blood meals, might be a response to the presence of trypanosomes, possibly through the production of anti-trypanosome products that are of oxidative nature. The enzyme lectizyme, which is involved in the establishment of trypanosome infection in tsetse flies, is described to have both lectin and protease activity; it may also be implicated in D-glucosamine binding, agglutination of the bloodstream form of trypanosomes, and in inducing the transformation of the bloodstream form into the procyclic form *in vitro* (Abubakar et al., 2006).

The identification of several proteins related to the translational machinery, such as eukaryotic translation initiation factor 3, was an unexpected result. However, these proteins were down-regulated following trypanosome stimulation. This result might be surprising when compared to the data reported by Nandan et al. (2002). However, some of these proteins, including elongation factor 1α, produced by *Leishmania* itself, is associated with the pathogen survival (Nandan et al., 2002).

Cytochrome oxidase was also down-regulated in flies fed an infected bloodmeal. Effects of cytochrome oxidase have been observed between schistosomes and their intermediate hosts. For example, alterations in cytochrome-c oxidase expression were observed between praziquantel-resistant and susceptible strains of *Schistosoma mansoni* (Pereira et al., 1998) while over-expressed mRNA was observed in resistant strain. In the case of regulating gene expression in tsetse flies after invasion by trypanosomes, cytochrome c oxidase down-regulation could interfere with the trypanosome apoptotic program, thereby allowing trypanosome development.

The enzyme Δ(1)-pyrroline-5-carboxylate (P5C) dehydrogenase is involved in the proline metabolic pathway, and was down-regulated in trypanosome-stimulated flies. A protein displaying the same catalytic activity was previously reported to be encoded by *W. glossinidia*, the tsetse fly obligate symbiont (Hamidou Soumana et al., 2014b). Furthermore, the expression of this *Wigglesworthia* gene was down-regulated when the symbiont was harbored by a tsetse fly which had ingested a trypanosome-infected blood meal (3 days post-feeding). Proline plays diverse and crucial roles, notably as a molecule whose oxidation by the proline oxidase-FAD complex delivers electrons to the electron transport chain and to O_2_, resulting in the overproduction of reactive oxygen species. Down-regulation of the tsetse P5C following an infected bloodmeal may affect the production of proline-rich proteins like tsetse EP that are involved in immunity (Haines et al., 2010) and the nutritional requirements of trypanosomes and bacteria symbionts.

GDP-mannose pyrophosphorylase (GDPMP) was also down-regulated in stimulated flies. It is widely accepted that the biosynthesis of mannose-containing glycoconjugates is vital for eukaryotic organisms (Varki, 1999). GDPMP is essential for GDP-mannose production; deletion of the gene encoding this enzyme is lethal in fungi, most likely as a consequence of disrupted glycoconjugate biosynthesis. Interestingly, the loss of GDPMP renders *Leishmania mexicana* unable to infect macrophages, whereas gene addition restores its virulence (Garami and Iig, 2001). However, this enzyme is part of a specialized pathway used by *Leishmania* for the production of mannosylated surface virulence factors, and does not exist in either insects or African trypanosomes. Thus, although glycosylation mechanisms are largely unknown in *Glossina* and insects, it is likely that the GDPMP function in *Glossina* is related to the expression of other mannosylated glycans, such as those from glycosylphosphatidylinositol (GPI) anchors and N-glycans (Savage et al., 2012). GPI-anchored proteins in mammalian systems have previously been described (or predicted) to have hydrolytic activity, or to serve as either receptors or adhesion molecules; roles for several of these proteins have also been suggested in trans-membrane signaling or membrane trafficking (Ferguson, 1999; Chatterjee and Mayor, 2001). Protein N-glycosylation in eukaryotes covers a wide range of functions including signaling (through interaction with lectins), protein stabilization, protease resistance, endocytic sorting functions, and protein folding (Helenius and Aebi, 2004).

Other proteins were down-regulated in flies fed on infected bloodmeal, such as glycolytic enzymes. Infection usually stimulates glycolytic enzyme production in cultured cells infected with viruses (Klemperer, 1961; El-Bacha et al., 2004) or in midguts of *Aedes aegypti* infected with chikungunya and dengue-2 viruses (Tchankouo-Nguetcheu et al., 2010). Moreover, recent studies have demonstrated that glycolytic enzymes may have alternative functions involved in transcriptional regulation, and may be regulators or indicators of apoptosis (Kim and Dang, 2005). The glycolytic enzyme GAPDH is also involved in the energetic metabolism of bloodstream trypanosomes, and can be considered as a virulence factor. One recent study has suggested that secreted GAPDH may be involved in virulence processes through an interaction with plasminogen and fibrinogen (Egea et al., 2007). Finally, coagulation deregulation processed by GAPDH could participate in the pathogenic effects induced by *T. congolense* in infected animals. However, it is unlikely that all of these processes occur in trypanosome-stimulated flies, since glycolytic enzymes were shown to be down-regulated 3 days after fly challenge with trypanosomes. This down-regulation could allow the trypanosome to evade the weakened defenses of tsetse flies and prepare their establishment in the insect midgut. Even if the ratios of up- and down-regulated proteins are low, the significant *p*-value observed is highly suggestive of a biologically meaningful variation.

A very limited number of the bacterial proteins (from *Wigglesworthia* and *Sodalis*) present at day 3 post-bloodmeal have been identified (Table 3), despite previous transcriptomic investigations that demonstrated high gene expression activity. The fact that we did not observe other symbiont proteins may be due to the large number of identified tsetse proteins (and proteins from the ingested mouse blood), which could conceal proteins present at extremely low concentrations. This situation is proof for the need to couple proteomics with specific transcriptomic studies.

## Conclusion

We have performed a molecular characterization of the differentially expressed proteins in the tsetse midgut 3 days after ingesting an infected vs. non-infected meal. The results provide a wide array of new findings that will improve our understanding of the effects induced by trypanosome invasion. In the context of our results, these proteins deserve specific attention with regard to their functions and the biological processes they are involved in. Further, investigations at the transcriptomic level will provide additional and valuable information on the regulation of genes that encode these proteins.

Furthermore, these results will enable the development of novel means to interfere with the infection process. Nevertheless, functional experiments (e.g., synthesis of native recombinant proteins and RNA interference) will be required to identify the most relevant candidates resulting from this proteomic approach. To be effective, these candidates must demonstrate that their expression modulation is capable of modifying the fly trypanosome-infection process, as well as fly vector competence.

### Conflict of interest statement

The authors declare that the research was conducted in the absence of any commercial or financial relationships that could be construed as a potential conflict of interest.
