# Supplementary material for: Differential expression of midgut proteins in Trypanosoma brucei gambiense-stimulated vs. non-stimulated Glossina palpalis gambiensis flies
Source: Front Microbiol. 2015 May 12;6:444. doi: 10.3389/fmicb.2015.00444 (PMC4428205; doi:10.3389/fmicb.2015.00444)
Supplement: Supplementary file 9 [file Table9.PDF]

Supplementary Table S9. Listing of significantly (p≤ 0.05 / Student t-test) up- and down-regulated proteins in stimulated versus non-stimulated flies.

Accession	Description	Start_Prot	End_Prot	Sequence	Unicity	Charge	Modification	Score_A	Score_B	MSurZ_A	MSurZ_B	Nbr_Id_Mascot_A	Nbr_Id_Mascot_B	Nbr_Int_Iq_A	Nbr_Int_Iq_B	Num_Replicate	Intensity_A	Intensity_A_norm	Intensity_B	Intensity_B_norm	Mean peptide A	Mean peptide B	T-Test data normalised	Ratio (B/A)
B4GFZ8, Q0QHL1	GL21529 OS=Drosophila persimilis GN=DperGL21529 PE=3 SV=1	47	57	TDIPSAQYGGR	3	2		0	0	0	0	0	0	1	1	1	43102,16	4,79295E-05	124024,27	0,000145656	5,79E-05	0,000124	0,0333204	2,13501
B4GFZ8, Q0QHL1	GL21529 OS=Drosophila persimilis GN=DperGL21529 PE=3 SV=1	47	57	TDIPSAQYGGR	3	2		0	0	0	0	0	0	1	1	2	23424,73	2,72795E-05	125141,67	0,000142094				
B4GFZ8, Q0QHL1	GL21529 OS=Drosophila persimilis GN=DperGL21529 PE=3 SV=1	47	57	TDIPSAQYGGR	3	2		46,49	0	582,7859	0	1	0	1	1	3	94690,37	9,84717E-05	88398,08	0,000107272				
B4GFZ8, Q0QHL1	GL21529 OS=Drosophila persimilis GN=DperGL21529 PE=3 SV=1	47	57	TDIPSAQYGGR	3	2		0	0	0	0	0	0	0	1	4	0	NA	80415,87	9,93904E-05				
B4NN02	GK22983 OS=Drosophila willistoni GN=DwilGK22983 PE=3 SV=1	100	113	LHFESGVIFEGALR	1	3		0	0	0	0	0	0	1	1	1	7843,19	8,72161E-06	11375,72	1,33598E-05	5,78E-06	1,1E-05	0,0338203	1,90522
B4NN02	GK22983 OS=Drosophila willistoni GN=DwilGK22983 PE=3 SV=1	100	113	LHFESGVIFEGALR	1	3		0	46,44	0	525,6156	0	1	1	1	2	5273,96	6,14185E-06	8583,4	9,74617E-06				
B4NN02	GK22983 OS=Drosophila willistoni GN=DwilGK22983 PE=3 SV=1	100	113	LHFESGVIFEGALR	1	3		0	0	0	0	0	0	0	1	3	0	NA	8222,3	9,97785E-06				
B4NN02	GK22983 OS=Drosophila willistoni GN=DwilGK22983 PE=3 SV=1	100	113	LHFESGVIFEGALR	1	3		0	0	0	0	0	0	1	1	4	1818,73	2,4733E-06	8864,98	1,09567E-05				
Q8MUG0	Lectizyme OS=Glossina fuscipes fuscipes GN=Gpl PE=2 SV=1	145	157	VNLPTGKYESTGK	1	3		0	61,01	0	465,2498	0	1	1	1	1	65069,21	7,23568E-05	63548,25	7,46321E-05	5,53E-05	8,61E-05	0,0090677	1,55514
Q8MUG0	Lectizyme OS=Glossina fuscipes fuscipes GN=Gpl PE=2 SV=1	145	157	VNLPTGKYESTGK	1	3		0	0	0	0	0	0	1	1	2	43584,25	5,07565E-05	74097,06	8,41348E-05				
Q8MUG0	Lectizyme OS=Glossina fuscipes fuscipes GN=Gpl PE=2 SV=1	145	157	VNLPTGKYESTGK	1	3		0	0	0	0	0	0	1	1	3	39306,05	4,08757E-05	80139,98	9,72507E-05				
Q8MUG0	Lectizyme OS=Glossina fuscipes fuscipes GN=Gpl PE=2 SV=1	145	157	VNLPTGKYESTGK	1	3		0	0	0	0	0	0	1	1	4	42206,5	5,73968E-05	71417,91	8,82693E-05				
D3TMN6	Eukaryotic translation initiation factor 3 subunit M OS=Glossina morsit	301	318	ELQISEDEVEPFVIEVLK	1	2		49,32	0	1058,5522	0	1	0	1	1	1	10580,83	1,17659E-05	2660,73	3,1248E-06	1,11E-05	2,02E-06	0,0269735	0,18203
D3TMN6	Eukaryotic translation initiation factor 3 subunit M OS=Glossina morsit	301	318	ELQISEDEVEPFVIEVLK	1	2		45,56	0	1058,5532	0	1	0	0	1	2	0	NA	1470,37	1,66956E-06				
D3TMN6	Eukaryotic translation initiation factor 3 subunit M OS=Glossina morsit	301	318	ELQISEDEVEPFVIEVLK	1	2		58,5	0	1058,5524	0	1	0	1	1	3	4793,62	4,98505E-06	2594,83	3,14885E-06				
D3TMN6	Eukaryotic translation initiation factor 3 subunit M OS=Glossina morsit	301	318	ELQISEDEVEPFVIEVLK	1	2		0	0	0	0	0	0	1	1	4	12173,3	1,65545E-05	113,35	1,40095E-07				
D3TSM2	Putative membrane protein OS=Glossina morsitans morsitans PE=2 SV	191	206	NTDTQDELEEVQSDLR	1	2		0	0	0	0	0	0	1	1	1	5494,36	6,10972E-06	733,85	8,61845E-07	8,23E-06	3,27E-06	0,0257971	0,3974
D3TSM2	Putative membrane protein OS=Glossina morsitans morsitans PE=2 SV	191	206	NTDTQDELEEVQSDLR	1	2		0	0	0	0	0	0	1	1	2	7323,59	8,52877E-06	4751,15	5,39478E-06				
D3TSM2	Putative membrane protein OS=Glossina morsitans morsitans PE=2 SV	191	206	NTDTQDELEEVQSDLR	1	2		63,44	0	946,4277	0	1	0	1	1	3	11572,03	1,20341E-05	2298,97	2,78982E-06				
D3TSM2	Putative membrane protein OS=Glossina morsitans morsitans PE=2 SV	191	206	NTDTQDELEEVQSDLR	1	2		0	0	0	0	0	0	1	1	4	4604,35	6,26148E-06	3270,09	4,04168E-06				
B4KZE6	GI12924 OS=Drosophila mojavensis GN=DmojGI12924 PE=4 SV=1	61	66	QPLISR	1	2		0	0	0	0	0	0	0	0	1	0	NA	0	NA	5,39E-05	2,29E-05	0,0327849	0,42475
B4KZE6	GI12924 OS=Drosophila mojavensis GN=DmojGI12924 PE=4 SV=1	61	66	QPLISR	1	2		0	48,34	0	357,2207	0	1	1	1	2	48669,42	5,66785E-05	36811,06	4,17978E-05				
B4KZE6	GI12924 OS=Drosophila mojavensis GN=DmojGI12924 PE=4 SV=1	61	66	QPLISR	1	2		0	48,55	0	357,2218	0	1	1	1	3	49582,05	5,1562E-05	9189,7	1,11518E-05				
B4KZE6	GI12924 OS=Drosophila mojavensis GN=DmojGI12924 PE=4 SV=1	61	66	QPLISR	1	2		0	0	0	0	0	0	1	1	4	39201,04	5,33097E-05	12677,11	1,56683E-05				
D3TSF3	Acyl-coenzyme A oxidase OS=Glossina morsitans morsitans PE=2 SV=1	426	440	AVCSADAASGVEVCR	1	2		55,6	83,78	776,3477	776,3467	3	2	1	1	1	57917,78	6,44045E-05	16497,97	1,93755E-05	8,89E-05	4,51E-05	0,0231591	0,5074
D3TSF3	Acyl-coenzyme A oxidase OS=Glossina morsitans morsitans PE=2 SV=1	426	440	AVCSADAASGVEVCR	1	2		73,14	51,39	776,3472	776,3471	1	2	1	1	2	87476,3	0,000101872	54124,32	6,14564E-05				
D3TSF3	Acyl-coenzyme A oxidase OS=Glossina morsitans morsitans PE=2 SV=1	426	440	AVCSADAASGVEVCR	1	2		82,8	48,48	776,3484	776,3466	3	2	1	1	3	108745,15	0,000113088	38239,04	4,64035E-05				
D3TSF3	Acyl-coenzyme A oxidase OS=Glossina morsitans morsitans PE=2 SV=1	426	440	AVCSADAASGVEVCR	1	2		57,63	66,25	776,3476	776,347	3	2	1	1	4	56063,95	7,62417E-05	43041,94	5,31979E-05				
D3TRY9	Cytochrome c oxidase subunit IV COX5b OS=Glossina morsitans morsit	97	114	ASFCQTFAEIQAPTGEFK	1	2		0	46,21	0	1016,4772	0	1	1	1	1	74491,68	8,28346E-05	72370,19	8,49927E-05	8,26E-05	4,35E-05	0,0411688	0,52715
D3TRY9	Cytochrome c oxidase subunit IV COX5b OS=Glossina morsitans morsit	97	114	ASFCQTFAEIQAPTGEFK	1	2		0	44,79	0	1016,4785	0	1	1	1	2	59217,52	6,89624E-05	22471,99	2,55162E-05				
D3TRY9	Cytochrome c oxidase subunit IV COX5b OS=Glossina morsitans morsit	97	114	ASFCQTFAEIQAPTGEFK	1	2		55,22	54,85	1016,4787	1016,4777	4	1	1	1	3	77999,95	8,11148E-05	26210,77	3,18071E-05				
D3TRY9	Cytochrome c oxidase subunit IV COX5b OS=Glossina morsitans morsit	97	114	ASFCQTFAEIQAPTGEFK	1	2		78,53	45,92	1016,4783	1016,4776	4	1	1	1	4	71653,3	9,74417E-05	25753,36	3,183E-05				
D3TQT6	Glycerol 3 phosphate dehydrogenase OS=Glossina morsitans morsitan	181	189	DLFQSENFR	1	2		0	46,52	0	578,2744	0	1	1	1	1	84100,77	9,35199E-05	107692,21	0,000126475	0,000149	7,46E-05	0,0308732	0,58554
D3TQT6	Glycerol 3 phosphate dehydrogenase OS=Glossina morsitans morsitan	181	189	DLFQSENFR	1	2		0	0	0	0	0	0	1	1	2	171015,67	0,000199158	90096,55	0,000102302				
D3TQT6	Glycerol 3 phosphate dehydrogenase OS=Glossina morsitans morsitan	181	189	DLFQSENFR	1	2		0	46,99	0	578,2754	0	1	1	1	3	181884,94	0,000189148	29634,56	3,59619E-05				
D3TQT6	Glycerol 3 phosphate dehydrogenase OS=Glossina morsitans morsitan	181	189	DLFQSENFR	1	2		0	0	0	0	0	0	1	1	4	82978,81	0,000112843	27217,72	3,36399E-05				
D3TQT6	Glycerol 3 phosphate dehydrogenase OS=Glossina morsitans morsitan	22	36	IVGANCAALPEFEDR	1	2		53,49	0	831,399	0	2	0	1	1	1	28031,7	3,11712E-05	33105,29	3,88794E-05	4,06E-05	2,58E-05		
D3TQT6	Glycerol 3 phosphate dehydrogenase OS=Glossina morsitans morsitan	22	36	IVGANCAALPEFEDR	1	2		54,6	0	831,4004	0	1	0	1	1	2	39881,78	4,64448E-05	20323,19	2,30763E-05				
D3TQT6	Glycerol 3 phosphate dehydrogenase OS=Glossina morsitans morsitan	22	36	IVGANCAALPEFEDR	1	2		49,18	53,86	831,4016	831,4005	1	1	1	1	3	50026,22	5,20239E-05	14896,52	1,80771E-05				
D3TQT6	Glycerol 3 phosphate dehydrogenase OS=Glossina morsitans morsitan	22	36	IVGANCAALPEFEDR	1	2		44,38	0	831,4004	0	1	0	1	1	4	24220,37	3,29374E-05	18649,96	2,30505E-05				
D3TQT6	Glycerol 3 phosphate dehydrogenase OS=Glossina morsitans morsitan	51	63	LTEIINTSHENVK	1	2		76,46	63,82	749,3978	749,3994	2	1	1	1	1	26088,79	2,90107E-05	33390,98	3,92149E-05	6,01E-05	3,42E-05		
D3TQT6	Glycerol 3 phosphate dehydrogenase OS=Glossina morsitans morsitan	51	63	LTEIINTSHENVK	1	2		57,37	67,2	749,3981	749,3986	1	2	1	1	2	67884,65	7,90558E-05	40581,96	4,60795E-05				
D3TQT6	Glycerol 3 phosphate dehydrogenase OS=Glossina morsitans morsitan	51	63	LTEIINTSHENVK	1	2		63,84	58,44	749,3986	749,398	1	1	1	1	3	90790,51	9,44161E-05	22254,42	2,7006E-05				
D3TQT6	Glycerol 3 phosphate dehydrogenase OS=Glossina morsitans morsitan	51	63	LTEIINTSHENVK	1	2		75,08	52,69	749,3993	749,3987	2	2	1	1	4	27925,38	3,79759E-05	19723,28	2,43771E-05				
D3TQT6	Glycerol 3 phosphate dehydrogenase OS=Glossina morsitans morsitan	178	189	TLRDLFQSENFR	1	3		0	0	0	0	0	0	1	1	1	30173,55	3,35529E-05	51284,61	6,02295E-05	0,000117	8,26E-05		
D3TQT6	Glycerol 3 phosphate dehydrogenase OS=Glossina morsitans morsitan	178	189	TLRDLFQSENFR	1	3		0	0	0	0	0	0	1	1	2	136050,72	0,000158439	97488,12	0,000110695				
D3TQT6	Glycerol 3 phosphate dehydrogenase OS=Glossina morsitans morsitan	178	189	TLRDLFQSENFR	1	3		0	0	0	0	0	0	1	1	3	166706,39	0,000173364	80049,13	9,71405E-05				
D3TQT6	Glycerol 3 phosphate dehydrogenase OS=Glossina morsitans morsitan	178	189	TLRDLFQSENFR	1	3		43,65	0	509,2642	0	1	0	1	1	4	75055,27	0,000102068	50525,7	6,24475E-05				
D3TQT6	Glycerol 3 phosphate dehydrogenase OS=Glossina morsitans morsitan	190	206	VVVVQDSDAVEICGALK	1	2		66,12	94,53	901,4705	901,4697	2	3	1	1	1	24413,17	2,71474E-05	15630,75	1,8357E-05	3,94E-05	2,04E-05		
D3TQT6	Glycerol 3 phosphate dehydrogenase OS=Glossina morsitans morsitan	190	206	VVVVQDSDAVEICGALK	1	2		61,18	81,66	901,4718	901,4712	2	3	1	1	2	33296,03	3,87753E-05	23403,14	2,65735E-05				
D3TQT6	Glycerol 3 phosphate dehydrogenase OS=Glossina morsitans morsitan	190	206	VVVVQDSDAVEICGALK	1	2		66,68	63,26	901,4702	901,471	2	2	1	1	3	50471,28	5,24868E-05	9882,14	1,19921E-05				
D3TQT6	Glycerol 3 phosphate dehydrogenase OS=Glossina morsitans morsitan	190	206	VVVVQDSDAVEICGALK	1	2		75,61	63,85	901,4717	901,4708	3	1	1	1	4	28875,15	3,92675E-05	19940,18	2,46452E-05				
D3TLS8	4-hydroxybutyrate coenzyme A transferase OS=Glossina morsitans mc	60	74	SGDTVFTSGAAATPK	1	2		45,19	0	705,3483	0	1	0	1	1	1	34542,68	3,84114E-05	36174,56	4,2484E-05	5,16E-05	3,3E-05	0,0473816	0,64023
D3TLS8	4-hydroxybutyrate coenzyme A transferase OS=Glossina morsitans mc	60	74	SGDTVFTSGAAATPK	1	2		50,02	50,26	705,3478	705,348	1	1	1	1	2	54246,7	6,31736E-05	28545,56	3,24126E-05				
D3TLS8	4-hydroxybutyrate coenzyme A transferase OS=Glossina morsitans mc	60	74	SGDTVFTSGAAATPK	1	2		48,68	48,73	705,3486	705,3473	2	1	1	1	3	59602,17	6,19823E-05	19763,77	2,39835E-05				
D3TLS8	4-hydroxybutyrate coenzyme A transferase OS=Glossina morsitans mc	60	74	SGDTVFTSGAAATPK	1	2		51,1	0	705,3478	0	2	0	1	1	4	31353,91	4,26383E-05	26812,52	3,31391E-05				
D3TRV5	Eukaryotic translation initiation factor 3 subunit I OS=Glossina morsita	129	142	TVDATTADQSPILR	1	2		60,1	49,43	744,3881	744,3885	1	2	1	1	1	24796,33	2,75735E-05	16730,41	1,96485E-05	2,73E-05	1,8E-05	0,0073104	0,66147
D3TRV5	Eukaryotic translation initiation factor 3 subunit I OS=Glossina morsita	129	142	TVDATTADQSPILR	1	2		76,23	79,64	744,3873	744,3863	3	2	1	1	2	19932,61	2,32127E-05	14971,89	1,70001E-05				
D3TRV5	Eukaryotic translation initiation factor 3 subunit I OS=Glossina morsita	129	142	TVDATTADQSPILR	1	2		43,83	89,6	744,3864	744,3874	1	1	1	1	3	24169,83	2,5135E-05	15903,55	1,92991E-05				
D3TRV5	Eukaryotic translation initiation factor 3 subunit I OS=Glossina morsita	129	142	TVDATTADQSPILR	1	2		70,86	58,98	744,388	744,3884	2	3	1	1	4	24406,41	3,31904E-05	13128,58	1,62263E-05				

Q0QHK6	1-pyrroline-5-carboxylate dehydrogenase 2 OS=Glossina morsitans mc	79	92	AGKEDVDLAVQAAR	1	2		64,85	57,51	721,883	721,8822	3	2	1	1	1	29347,25	3,26341E-05	46421,01	5,45176E-05	5,65E-05	3,75E-05	0,0201477	0,70679
Q0QHK6	1-pyrroline-5-carboxylate dehydrogenase 2 OS=Glossina morsitans mc	79	92	AGKEDVDLAVQAAR	1	2		59,16	86,82	721,8825	721,8826	1	4	1	1	2	65978,44	7,68359E-05	36821,72	4,18099E-05				
Q0QHK6	1-pyrroline-5-carboxylate dehydrogenase 2 OS=Glossina morsitans mc	79	92	AGKEDVDLAVQAAR	1	2		62,51	72,18	721,8837	721,8825	7	5	1	1	3	79438,03	8,26103E-05	22464,26	2,72606E-05				
Q0QHK6	1-pyrroline-5-carboxylate dehydrogenase 2 OS=Glossina morsitans mc	79	92	AGKEDVDLAVQAAR	1	2		94,99	72,43	721,8828	721,8817	7	2	1	1	4	25072,19	3,40958E-05	21399,67	2,6449E-05				
Q0QHK6	1-pyrroline-5-carboxylate dehydrogenase 2 OS=Glossina morsitans mc	79	92	AGKEDVDLAVQAAR	1	3		47,99	0	481,5919	0	1	0	1	1	1	32653,35	3,63105E-05	42654,43	5,00941E-05	4,95E-05	3,89E-05		
Q0QHK6	1-pyrroline-5-carboxylate dehydrogenase 2 OS=Glossina morsitans mc	79	92	AGKEDVDLAVQAAR	1	3		0	55,93	0	481,5916	0	1	1	1	2	52947,14	6,16602E-05	46437,77	5,27286E-05				
Q0QHK6	1-pyrroline-5-carboxylate dehydrogenase 2 OS=Glossina morsitans mc	79	92	AGKEDVDLAVQAAR	1	3		68,18	0	481,5918	0	2	0	0	0	3	0	NA	0	NA				
Q0QHK6	1-pyrroline-5-carboxylate dehydrogenase 2 OS=Glossina morsitans mc	79	92	AGKEDVDLAVQAAR	1	3		46,28	0	481,5918	0	1	0	1	1	4	37194,74	5,05813E-05	11173,36	1,38098E-05				
Q0QHK6	1-pyrroline-5-carboxylate dehydrogenase 2 OS=Glossina morsitans mc	464	472	ANYIVQGLR	1	2		0	0	0	0	0	0	1	1	1	56681,56	6,30298E-05	49935,01	5,86445E-05	8,3E-05	9,41E-05		
Q0QHK6	1-pyrroline-5-carboxylate dehydrogenase 2 OS=Glossina morsitans mc	464	472	ANYIVQGLR	1	2		0	0	0	0	0	0	0	1	2	0	NA	115759,3	0,000131441				
Q0QHK6	1-pyrroline-5-carboxylate dehydrogenase 2 OS=Glossina morsitans mc	464	472	ANYIVQGLR	1	2		48,45	0	517,2892	0	1	0	1	0	3	101442,14	0,000105493	0	NA				
Q0QHK6	1-pyrroline-5-carboxylate dehydrogenase 2 OS=Glossina morsitans mc	464	472	ANYIVQGLR	1	2		0	0	0	0	0	0	1	1	4	59110,59	8,03848E-05	74534,94	9,21218E-05				
Q0QHK6	1-pyrroline-5-carboxylate dehydrogenase 2 OS=Glossina morsitans mc	460	472	DIDKANYIVQGLR	1	2		50,89	0	752,9101	0	2	0	1	1	1	50070,85	5,56787E-05	48133,08	5,65283E-05	0,000149	4,2E-05		
Q0QHK6	1-pyrroline-5-carboxylate dehydrogenase 2 OS=Glossina morsitans mc	460	472	DIDKANYIVQGLR	1	2		49,04	53,22	752,9093	752,9092	2	5	1	1	2	61875,68	7,2058E-05	24732,44	2,80829E-05				
Q0QHK6	1-pyrroline-5-carboxylate dehydrogenase 2 OS=Glossina morsitans mc	460	472	DIDKANYIVQGLR	1	2		62,51	61,04	752,9096	752,9093	6	5	1	1	3	350822,75	0,000364832	15668,47	1,90139E-05				
Q0QHK6	1-pyrroline-5-carboxylate dehydrogenase 2 OS=Glossina morsitans mc	460	472	DIDKANYIVQGLR	1	2		64,78	56,93	752,9095	752,9099	5	4	1	1	4	76832,17	0,000104484	52246,35	6,45741E-05				
Q0QHK6	1-pyrroline-5-carboxylate dehydrogenase 2 OS=Glossina morsitans mc	423	434	EEIFGPVQQIIR	3	2		0	0	0	0	0	0	1	1	1	47072,28	5,23443E-05	14394,91	1,69056E-05	7,57E-05	2,15E-05		
Q0QHK6	1-pyrroline-5-carboxylate dehydrogenase 2 OS=Glossina morsitans mc	423	434	EEIFGPVQQIIR	3	2		0	0	0	0	0	0	1	1	2	82146,35	9,56645E-05	25970,1	2,94882E-05				
Q0QHK6	1-pyrroline-5-carboxylate dehydrogenase 2 OS=Glossina morsitans mc	423	434	EEIFGPVQQIIR	3	2		43,53	0	714,8961	0	1	0	1	1	3	114962,47	0,000119553	7221,13	8,76292E-06				
Q0QHK6	1-pyrroline-5-carboxylate dehydrogenase 2 OS=Glossina morsitans mc	423	434	EEIFGPVQQIIR	3	2		48,14	0	714,8961	0	4	0	1	1	4	26023,97	3,53901E-05	25110,46	3,10354E-05				
Q0QHK6	1-pyrroline-5-carboxylate dehydrogenase 2 OS=Glossina morsitans mc	420	434	IAREEIFGPVQQIIR	1	3		54,47	50,64	590,3414	590,3407	2	2	1	1	1	79704,86	8,86317E-05	130187,16	0,000152894	0,000189	0,000146		
Q0QHK6	1-pyrroline-5-carboxylate dehydrogenase 2 OS=Glossina morsitans mc	420	434	IAREEIFGPVQQIIR	1	3		51	57,87	590,3398	590,3414	2	6	1	1	2	207274,93	0,000241384	140837,44	0,000159916				
Q0QHK6	1-pyrroline-5-carboxylate dehydrogenase 2 OS=Glossina morsitans mc	420	434	IAREEIFGPVQQIIR	1	3		74,08	50,08	590,3402	590,3406	11	5	1	1	3	276614,51	0,000287661	123355,76	0,000149694				
Q0QHK6	1-pyrroline-5-carboxylate dehydrogenase 2 OS=Glossina morsitans mc	420	434	IAREEIFGPVQQIIR	1	3		69,94	55,65	590,3402	590,3401	14	4	1	1	4	103135,67	0,000140255	98028,23	0,000121158				
Q0QHK6	1-pyrroline-5-carboxylate dehydrogenase 2 OS=Glossina morsitans mc	377	390	ILQLIDSGKQQGAK	1	2		0	0	0	0	0	0	1	1	1	23921,9	2,66011E-05	33937,7	3,9857E-05	4,36E-05	2,5E-05		
Q0QHK6	1-pyrroline-5-carboxylate dehydrogenase 2 OS=Glossina morsitans mc	377	390	ILQLIDSGKQQGAK	1	2		0	53,27	0	749,9326	0	2	1	1	2	40963,68	4,77047E-05	25975,86	2,94948E-05				
Q0QHK6	1-pyrroline-5-carboxylate dehydrogenase 2 OS=Glossina morsitans mc	377	390	ILQLIDSGKQQGAK	1	2		51,23	0	749,9322	0	1	0	1	1	3	63983,83	6,65389E-05	17305,55	2,10005E-05				
Q0QHK6	1-pyrroline-5-carboxylate dehydrogenase 2 OS=Glossina morsitans mc	377	390	ILQLIDSGKQQGAK	1	2		63,88	0	749,9324	0	4	0	1	1	4	24646,11	3,35164E-05	7820,73	9,66607E-06				
Q0QHK6	1-pyrroline-5-carboxylate dehydrogenase 2 OS=Glossina morsitans mc	377	390	ILQLIDSGKQQGAK	1	3		57,85	0	500,2914	0	1	0	1	1	1	76296,4	8,48415E-05	59546,97	6,99329E-05	0,000107	5,8E-05		
Q0QHK6	1-pyrroline-5-carboxylate dehydrogenase 2 OS=Glossina morsitans mc	377	390	ILQLIDSGKQQGAK	1	3		0	43,94	0	500,291	0	1	1	1	2	74482,96	8,674E-05	72769,36	8,26273E-05				
Q0QHK6	1-pyrroline-5-carboxylate dehydrogenase 2 OS=Glossina morsitans mc	377	390	ILQLIDSGKQQGAK	1	3		44,81	67,55	500,2906	500,2906	1	2	1	1	3	157567,79	0,00016386	23505,58	2,85243E-05				
Q0QHK6	1-pyrroline-5-carboxylate dehydrogenase 2 OS=Glossina morsitans mc	377	390	ILQLIDSGKQQGAK	1	3		0	0	0	0	0	0	1	1	4	68504,44	9,31596E-05	41060,63	5,07491E-05				
Q0QHK6	1-pyrroline-5-carboxylate dehydrogenase 2 OS=Glossina morsitans mc	276	287	LIQQASGNTNLK	1	2		64,93	0	643,8551	0	1	0	1	1	1	91416,15	0,000101655	116322,83	0,000136611	0,000131	0,000131		
Q0QHK6	1-pyrroline-5-carboxylate dehydrogenase 2 OS=Glossina morsitans mc	276	287	LIQQASGNTNLK	1	2		0	58,25	0	643,8562	0	2	1	1	2	154535,96	0,000179967	150815,33	0,000171246				
Q0QHK6	1-pyrroline-5-carboxylate dehydrogenase 2 OS=Glossina morsitans mc	276	287	LIQQASGNTNLK	1	2		74,97	65,12	643,8563	643,8558	5	5	1	1	3	166826,83	0,000173489	116633,57	0,000141536				
Q0QHK6	1-pyrroline-5-carboxylate dehydrogenase 2 OS=Glossina morsitans mc	276	287	LIQQASGNTNLK	1	2		77,86	52,53	643,8559	643,8557	3	2	1	1	4	49205,25	6,69145E-05	59824,94	7,39409E-05				
Q0QHK6	1-pyrroline-5-carboxylate dehydrogenase 2 OS=Glossina morsitans mc	276	288	LIQQASGNTNLKR	1	2		0	45,41	0	721,9062	0	1	1	1	1	29080,76	3,23378E-05	56071,19	6,58509E-05	6,07E-05	5,86E-05		
Q0QHK6	1-pyrroline-5-carboxylate dehydrogenase 2 OS=Glossina morsitans mc	276	288	LIQQASGNTNLKR	1	2		0	0	0	0	0	0	1	1	2	46418,7	5,40574E-05	64540,58	7,32838E-05				
Q0QHK6	1-pyrroline-5-carboxylate dehydrogenase 2 OS=Glossina morsitans mc	276	288	LIQQASGNTNLKR	1	2		67,85	49,81	721,9059	721,9067	1	1	1	1	3	107645,92	0,000111945	40993,97	4,97466E-05				
Q0QHK6	1-pyrroline-5-carboxylate dehydrogenase 2 OS=Glossina morsitans mc	276	288	LIQQASGNTNLKR	1	2		47,04	45,33	721,9066	721,9059	2	1	1	1	4	32685,62	4,44493E-05	36711,1	4,53733E-05				
Q0QHK6	1-pyrroline-5-carboxylate dehydrogenase 2 OS=Glossina morsitans mc	276	288	LIQQASGNTNLKR	1	3		73,36	70,25	481,6076	481,6078	5	3	1	1	1	134082,31	0,000149099	182459,58	0,000214284	0,00024	0,000196		
Q0QHK6	1-pyrroline-5-carboxylate dehydrogenase 2 OS=Glossina morsitans mc	276	288	LIQQASGNTNLKR	1	3		76,36	62,9	481,6073	481,608	7	5	1	1	2	262138,05	0,000305276	239484,26	0,000271927				
Q0QHK6	1-pyrroline-5-carboxylate dehydrogenase 2 OS=Glossina morsitans mc	276	288	LIQQASGNTNLKR	1	3		84,7	75,97	481,608	481,6075	12	14	1	1	3	378630,26	0,00039375	150381,11	0,000182489				
Q0QHK6	1-pyrroline-5-carboxylate dehydrogenase 2 OS=Glossina morsitans mc	276	288	LIQQASGNTNLKR	1	3		77,16	73,86	481,6083	481,6073	13	8	1	1	4	82956,67	0,000112813	94313,97	0,000116568				
Q0QHK6	1-pyrroline-5-carboxylate dehydrogenase 2 OS=Glossina morsitans mc	61	71	TFPSINPTTEK	1	2		0	0	0	0	0	0	1	1	1	47816,71	5,31721E-05	103387,2	0,00012142	7,68E-05	7,71E-05		
Q0QHK6	1-pyrroline-5-carboxylate dehydrogenase 2 OS=Glossina morsitans mc	61	71	TFPSINPTTEK	1	2		0	0	0	0	0	0	1	1	2	124000,39	0,000144406	78191,79	8,87843E-05				
Q0QHK6	1-pyrroline-5-carboxylate dehydrogenase 2 OS=Glossina morsitans mc	61	71	TFPSINPTTEK	1	2		0	0	0	0	0	0	1	1	3	79539,28	8,27156E-05	46225,84	5,60956E-05				
Q0QHK6	1-pyrroline-5-carboxylate dehydrogenase 2 OS=Glossina morsitans mc	61	71	TFPSINPTTEK	1	2		44,63	0	617,8183	0	1	0	1	1	4	19900,22	2,70624E-05	34215,52	4,22888E-05				
Q0QHK6	1-pyrroline-5-carboxylate dehydrogenase 2 OS=Glossina morsitans mc	168	180	TIPMDGDFFAYTR	1	2		0	0	0	0	0	0	1	1	1	23431,63	2,60559E-05	29577,02	3,47357E-05	3,77E-05	1,92E-05		
Q0QHK6	1-pyrroline-5-carboxylate dehydrogenase 2 OS=Glossina morsitans mc	168	180	TIPMDGDFFAYTR	1	2		0	0	0	0	0	0	1	1	2	35016,66	4,0779E-05	15076,81	1,71192E-05				
Q0QHK6	1-pyrroline-5-carboxylate dehydrogenase 2 OS=Glossina morsitans mc	168	180	TIPMDGDFFAYTR	1	2		0	0	0	0	0	0	1	1	3	41257,41	4,2905E-05	9082,86	1,10221E-05				
Q0QHK6	1-pyrroline-5-carboxylate dehydrogenase 2 OS=Glossina morsitans mc	168	180	TIPMDGDFFAYTR	1	2		60,55	0	767,3553	0	1	0	1	1	4	30068,52	4,08903E-05	11300,7	1,39672E-05				
Q0QHK6	1-pyrroline-5-carboxylate dehydrogenase 2 OS=Glossina morsitans mc	168	180	TIPMDGDFFAYTR	1	2	TIPM[Oxidation (M)]DG	0	0	0	0	0	0	1	1	1	48967,67	5,4452E-05	47314,54	5,5567E-05	0,000112	6,36E-05		
Q0QHK6	1-pyrroline-5-carboxylate dehydrogenase 2 OS=Glossina morsitans mc	168	180	TIPMDGDFFAYTR	1	2	TIPM[Oxidation (M)]DG	57,5	50,48	775,3549	775,3556	2	3	1	1	2	86973,4	0,000101286	86650,93	9,83893E-05				
Q0QHK6	1-pyrroline-5-carboxylate dehydrogenase 2 OS=Glossina morsitans mc	168	180	TIPMDGDFFAYTR	1	2	TIPM[Oxidation (M)]DG	55,56	0	775,3532	0	2	0	1	1	3	198692,69	0,000206627	32729,87	3,9718E-05				
Q0QHK6	1-pyrroline-5-carboxylate dehydrogenase 2 OS=Glossina morsitans mc	168	180	TIPMDGDFFAYTR	1	2	TIPM[Oxidation (M)]DG	53,06	55,65	775,3533	775,355	4	2	1	1	4	64326,29	8,74777E-05	49070,13	6,06485E-05				
Q0QHK6	1-pyrroline-5-carboxylate dehydrogenase 2 OS=Glossina morsitans mc	265	275	VAFTGSTEVGK	1	2		0	0	0	0	0	0	1	1	1	131676,4	0,000146424	196972,27	0,000231327	0,000225	0,000149		
Q0QHK6	1-pyrroline-5-carboxylate dehydrogenase 2 OS=Glossina morsitans mc	265	275	VAFTGSTEVGK	1	2		0	47,03	0	548,2878	0	1	1	1	2	189925,22	0,00022118	114778,96	0,000130328				
Q0QHK6	1-pyrroline-5-carboxylate dehydrogenase 2 OS=Glossina morsitans mc	265	275	VAFTGSTEVGK	1	2		53,1	0	548,2881	0	1	0	1	1	3	335017,62	0,000348396	101606,44	0,000123301				
Q0QHK6	1-pyrroline-5-carboxylate dehydrogenase 2 OS=Glossina morsitans mc	265	275	VAFTGSTEVGK	1	2		67,51	0	548,2881	0	2	0	1	1	4	136601,42	0,000185765	90472,13	0,000111819				
D3TND8	GDP-mannose pyrophosphorylase OS=Glossina morsitans morsitans P	254	271	LHSGPGIVGNVLVDPSAK	1	3		0	0	0	0	0	0	1	1	1	29580,69	3,28937E-05	26469,9	3,10867E-05	3,73E-05	2,72E-05	0,0200217	0,73083
D3TND8	GDP-mannose pyrophosphorylase OS=Glossina morsitans morsitans P	254	271	LHSGPGIVGNVLVDPSAK	1	3		0	59,73	0	587,3274	0	2	1	1	2	32140,91	3,74301E-05	25277,32	2,87016E-05				
D3TND8	GDP-mannose pyrophosphorylase OS=Glossina morsitans morsitans P	254	271	LHSGPGIVGNVLVDPSAK	1	3		0	0	0	0	0	0	1	1	3	41103,72	4,27451E-05	23915,25	2,90214E-05				
D3TND8	GDP-mannose pyrophosphorylase OS=Glossina morsitans morsitans P	254	271	LHSGPGIVGNVLVDPSAK	1	3		59,41	0	587,3279	0	2	0	1	1	4	26423,99	3,59341E-05	16251,6	2,00862E-05				
